# Semi-supervised protein subcellular localization

**DOI:** 10.1186/1471-2105-10-S1-S47

**Published:** 2009-01-30

**Authors:** Qian Xu, Derek Hao Hu, Hong Xue, Weichuan Yu, Qiang Yang

**Affiliations:** 1 Program of Bioengineering, Hong Kong University of Science and Technology, Clear Water Bay, Kowloon, Hong Kong; 2 Department of Computer Science and Engineering, Hong Kong University of Science and Technology, Clear Water Bay, Kowloon, Hong Kong; 3 Department of Biochemistry, Hong Kong University of Science and Technology, Clear Water Bay, Kowloon, Hong Kong; 4 Department of Electronic and Computer Engineering, Hong Kong University of Science and Technology, Clear Water Bay, Kowloon, Hong Kong

## Abstract

**Background:**

Protein subcellular localization is concerned with predicting the location of a protein within a cell using computational method. The location information can indicate key functionalities of proteins. Accurate predictions of subcellular localizations of protein can aid the prediction of protein function and genome annotation, as well as the identification of drug targets. Computational methods based on machine learning, such as support vector machine approaches, have already been widely used in the prediction of protein subcellular localization. However, a major drawback of these machine learning-based approaches is that a large amount of data should be labeled in order to let the prediction system learn a classifier of good generalization ability. However, in real world cases, it is laborious, expensive and time-consuming to experimentally determine the subcellular localization of a protein and prepare instances of labeled data.

**Results:**

In this paper, we present an approach based on a new learning framework, semi-supervised learning, which can use much fewer labeled instances to construct a high quality prediction model. We construct an initial classifier using a small set of labeled examples first, and then use unlabeled instances to refine the classifier for future predictions.

**Conclusion:**

Experimental results show that our methods can effectively reduce the workload for labeling data using the unlabeled data. Our method is shown to enhance the state-of-the-art prediction results of SVM classifiers by more than 10%.

## Background

**Figure 1:**
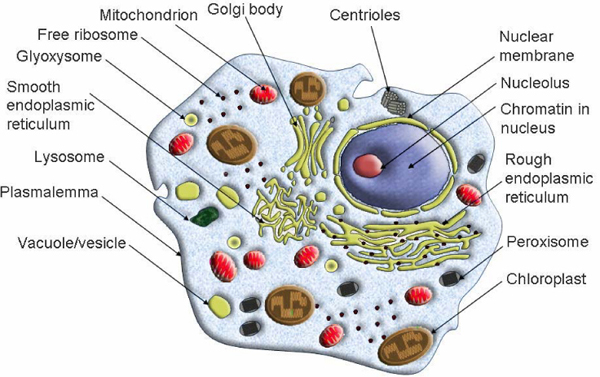
**Organelles with different functions in a cell**. This figure shows the organelles with different functions are the specialized subunits in a cell. Most organelles are closed compartments separated by lipid membranes, such as mitochondria, chloroplasts, peroxisomes, lysosomes, endoplasmic reticulum, cell nucleus and Golgi apparatus. Protein subcellular located within different organelles plays different role.

Organelles with different functions are the specialized subunits in a cell. (See Figure 1.) Most organelles are closed compartments separated by lipid membranes, such as mitochondria, chloroplasts, peroxisomes, lysosomes, endoplasmic reticulum, cell nucleus and Golgi apparatus. These compartments play different roles, for instance, mitochondria supply chemical energy ATP for cell survive; chloroplasts transform light energy to chemical energy using photosynthesis; peroxisomes participate metabolism process; lysosomes degrade engulfed viruses or bacteria, and destroyed organelles; cell nucleus contains almost genetic information, carried by DNA together with variable proteins to form chromosomes; Golgi apparatus is responsible to package proteins and lipids and modify chemicals to make them functional [1].

Protein subcellular localization is crucial for genome annotation, protein function prediction, and drug discovery [2]. Proteins perform their appropriate functions as, and only when, they localize in the correct subcellular compartments. Take prokaryotic and eukaryotic proteins as examples, for prokaryotes, many proteins that are synthesized in the cytoplasm are ultimately found noncytoplasmic locations [3], such as to a cell membrane or the extracellular environment, while most eukaryotic proteins are encoded in the nuclear and transported to the cytosol for further synthesis.

The annotations of protein subcellular localization can be detected by various wet-lab experiments. Cell fractionation, electron microscopy and fluorescence microscopy are three major experimental methods for the study of protein subcellular localization. However, the experimental approaches are time-consuming and expensive, so that there is a wide gap between the number of known protein subcellular localizations and the number of uncovered ones. For instance, according to the Swiss-Prot database version 50.0 related on 30-May-2006 the number of protein sequences with localization annotations is just about 14% of total eukaryotic protein entries [1]. This means that there are about 86% of eukaryotic protein entries without localization labels, which motivates us to find computational methods to predict the protein subcellular localization automatically and accurately.

Many methods have been developed and applied in an attempt to predict protein subcellular localization. Methods in [4-17] are based on amino acid composition to predict localization. Furthermore, scientists took account of sequence order together with amino acid composition to overcome information missing problem [18-21]. In addition, supervised learning algorithms such neural networks [22], K-NN algorithm [23], SVM [7,24,25] are widely applied to solve this problem. Among these learning-based approaches, SVM is popularly adopted in bioinformatics and is shown to perform relatively better compared to many others. There are also a large number of specialized databases are exploited such as DBSubLoc [26], ESLPred [27], HSLpred [28], LOCSVMPSI [29], LOC3d [30], PSORTb [31], PSORT [32], LOCtree [33], BaCelLo [34], TargetP [35], SecretomeP [36], PredictNLS [37], WoLF PSORT [38], Proteome Analyst [39], and CELLO [40].

In this paper, we present a novel approach to exploit the use of unlabeled data to aid the overall accuracy of protein subcellular localization and reduce the labeling effort. The existence of the relative large amount of unlabeled data provides us with a chance to mine useful information about the statistical distributions. We resort to two classical machine learning approaches, namely semi-supervised learning and ensemble learning. Experimental results on real biological data sets demonstrate that our efforts can effectively improve the accuracy of the state-of-the-art SVM classifiers with fewer labeled instances.

## Results and discussion

### Materials

This protein dataset includes 7,579 eukaryotic proteins with determined subcellular localizations, which were extracted from SWISS-PROT release 39.0 by Park and Kanehisa [41] and 34,521 eukaryotic proteins without subcellular localization information also extracted from SWISS-PROT. Within 7,579 proteins, there are 12 localizations: Chloroplast, Cytoplasmic, Cytoskeleton, Endoplasmic reticulum, Extracellular, Golgi apparatus, Lysosomal, Mitochondrial, Nuclear, Peroxisomal, Plasma membrane, Vacuolar. Detailed statistics of this protein dataset is shown in the following Table 1.

**Table 1:** Distribution of protein subcellular localizations

Subcellular Localizations	Number of proteins
Chloroplast	671
Cytoplasmic	1241
Cytoskeletion	40
Endoplasmic reticulum	114
Extracellular	861
Golgi apparatus	47
Lysosomal	93
Mitochondrial	727
Nuclear	1932
Peroxisomal	125
Plasma membrane	1674
Vacuolar	54

Total	7579

We adopt the 2-gram protein encoding method to generate feature of amino acid compositions, which is widely used in many existing protein subcellular localization protein systems [42].

### Empirical evaluations

We conducted extensive experiments to compare the CoForest approach with other state-of-the art prediction algorithms based on evaluation measurement 'accuracy'. In this paper, accuracy is defined as the proportion of true results, namely,

(1) $$Accuracy=\frac{TP+TN}{TP+FP+FN+TN}$$

TP means: the number of True Positives

TN means: the number of True Negatives

FP means: the number of False Positives

FN means: the number of False Negatives

#### Can we achieve same or better prediction with fewer labeled data?

We first demonstrate that our semi-supervised learning approach is indeed useful. In the next method section, we will demonstrate that two parameters will affect the overall performance of CoForest. We have chosen different values of *F *and *N *and also different numbers of labeled instances. The labeled instances are drawn randomly from the 12 localization classes in the labeled dataset. We sample the number of labeled instances from 1,000 to 3,000 and also change the number of classifiers from 60 to 200. As a result, the corresponding prediction accuracy on the whole set of 7,579 labeled instances are computed. The results in terms of prediction accuracy are described in Table 2, Table 3 and Table 4.

**Table 2:** Performance of Co-Forest Algorithm on the selected datasets(*F *= 20)

N	N = 60	N = 100	N = 150	N = 200
Labeled = 1000	0.5863	0.604	0.616	0.6289
Labeled = 1200	0.6285	0.6539	0.6716	0.6936
Labeled = 1400	0.6498	0.674	0.6907	0.7018
Labeled = 1600	0.703	0.7251	0.7352	0.7425
Labeled = 1800	0.7218	0.7441	0.7525	0.7589
Labeled = 2000	0.7416	0.769	0.78	0.7613
Labeled = 2200	0.7703	0.7898	0.7935	0.7627
Labeled = 2400	0.7896	0.8045	0.8125	0.8158
Labeled = 2600	0.8035	0.8181	0.8208	0.8209
Labeled = 2800	0.8162	0.8294	0.8318	0.8307
Labeled = 3000	0.83	0.834	0.847	0.8503

**Table 3:** Performance of Co-Forest Algorithm on the selected datasets(*F *= 40)

N	N = 60	N = 100	N = 150	N = 200
Labeled = 1000	0.5916	0.6114	0.6277	0.631
Labeled = 1200	0.6478	0.6656	0.6707	0.6818
Labeled = 1400	0.667	0.6919	0.6924	0.6962
Labeled = 1600	0.7071	0.726	0.7391	0.7416
Labeled = 1800	0.7215	0.7467	0.7502	0.7638
Labeled = 2000	0.7423	0.7598	0.7613	0.7707
Labeled = 2200	0.7715	0.7849	0.7913	0.8052
Labeled = 2400	0.7952	0.8054	0.8132	0.8208
Labeled = 2600	0.8092	0.8341	0.8244	0.8303
Labeled = 2800	0.8157	0.8312	0.8338	0.8394
Labeled = 3000	0.8298	0.8407	0.8525	0.8595

**Table 4:** Performance of Co-Forest Algorithm on the selected dataset (*F *= 60)

N	N = 60	N = 100	N = 150	N = 200
Labeled = 1000	0.5969	0.6226	0.6208	0.632
Labeled = 1200	0.6521	0.6736	0.6745	0.6928
Labeled = 1400	0.6637	0.6832	0.6933	0.7049
Labeled = 1600	0.7075	0.7228	0.7337	0.7483
Labeled = 1800	0.7351	0.7532	0.7591	0.7658
Labeled = 2000	0.7526	0.7618	0.7913	0.7956
Labeled = 2200	0.7736	0.7888	0.7873	0.8027
Labeled = 2400	0.7942	0.8099	0.8174	0.823
Labeled = 2600	0.812	0.8235	0.8252	0.8295
Labeled = 2800	0.8219	0.831	0.834	0.8367
Labeled = 3000	0.836	0.838	0.8493	0.8504

From the results, we can see that by using only about 20% of the labeled instances, we can achieve a prediction accuracy of more than 75%. As a rule of thumb, we can see that the prediction accuracy increases as *F *and *N *increase. This follows from our intuition of the algorithm description in the last section.

#### Comparison with baseline algorithms

We also compared CoForest with a number of machine learning algorithms, such as Decision Tree, AdaBoost and SVM. The reason for us to choose these classifiers as baseline algorithms are as follows: Since the weak learners we use in CoForest algorithm are in fact decision trees, we want to demonstrate the effectiveness of ensemble learning in our approach. Furthermore, since AdaBoost is also one of the most effective ensemble learning algorithms, we want to show that by using AdaBoost one could not achieve the same performance as our classifier does, where AdaBoost did not use unlabeled data to help refine the accuracy. A third choice of our baseline classifiers is the Support Vector Machine (SVM), which is the state-of-the-art algorithm in protein subcellular localization. We use this algorithm to show that our algorithm can perform better by using even fewer labeled instances.

For all the three baseline algorithms, we did not use any unlabeled instance since they are supervised machine learning algorithms and did not use the information from unlabeled data. We also ranged the number of training instances from 1,000 to 7,579 to show different levels of prediction accuracy as a function of labeled training data.

For decision tree, we used the C4.5 package implemented in Weka [43] and tested the algorithm accuracy in two settings. One setting is the ten-fold cross validation, where we randomly split the labeled data into ten folds, where one is used for testing and the other nine for training. This process is iterated ten times and the resulting ten classification accuracy values are averaged to get the final result of ten-fold cross validation. Another test setting is to simply use the whole set of 7,579 labeled instances for testing. For AdaBoost, we applied the AdaBoost package in Weka, and used decision stump as weak learners. Again we use 10-fold cross validation and external testing for the two test settings. Experimental results for these two baseline algorithms are shown in Table 5. We could see that by using only the tree-based approach on AdaBoost, the overall performance is relatively lower than the CoForest approach.

**Table 5:** Performance of baseline classifiers

Labeled Data	Tree(10-CV)	Tree(External)	AdaBoost(10-CV)	AdaBoost(External)
Labeled = 1000	0.256	0.3167	0.124	0.1124
Labeled = 1200	0.275	0.3628	0.1442	0.1989
Labeled = 1400	0.2485	0.3730	0.1943	0.2697
Labeled = 1600	0.2887	0.4350	0.1913	0.2488
Labeled = 1800	0.2772	0.4465	0.1917	0.2210
Labeled = 2000	0.2985	0.4749	0.2065	0.2702
Labeled = 2200	0.3222	0.5084	0.1868	0.2515
Labeled = 2400	0.2913	0.5296	0.1929	0.3544
Labeled = 2600	0.3173	0.5503	0.1981	0.2697
Labeled = 2800	0.3357	0.5601	0.2204	0.3524
Labeled = 3000	0.3387	0.5842	0.2160	0.3544
Labeled = 7579	0.4521	0.6234	0.3544	0.4041

**Figure 2:**
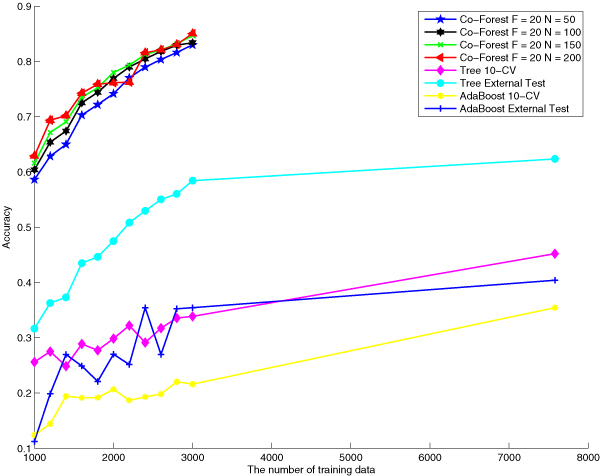
**Accuracy comparison of different approaches**. Accuracy comparison among Co-Forest F = 20, N = 100, Co-Forest F = 20, N = 150, Co-Forest F = 20, N = 200, decision tree with 10 cross validation, decision tree with external test, AdaBoost with 10 cross validation and AdaBoost with external test when training classifiers with different sample sizes.

**Figure 3:**
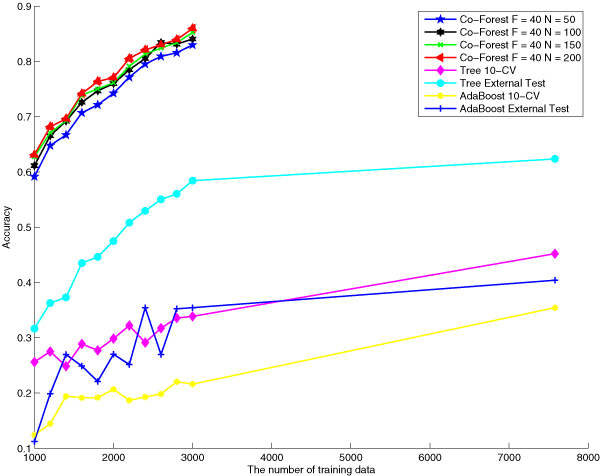
**Accuracy comparison of different approaches**. Accuracy comparison among Co-Forest F = 40, N = 100, Co-Forest F = 40, N = 150, Co-Forest F = 20, N = 200, decision tree with 10 cross validation, decision tree with external test, AdaBoost with 10 cross validation and AdaBoost with external test when training classifiers with different sample sizes.

**Figure 4:**
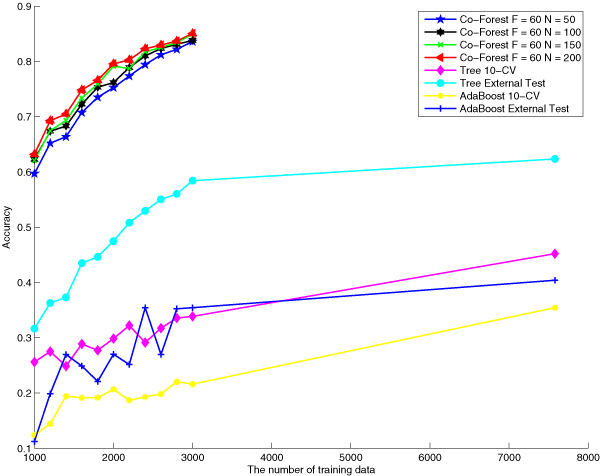
**Accuracy comparison of different approaches**. Accuracy comparison among Co-Forest F = 60, N = 100, Co-Forest F = 60, N = 150, Co-Forest F = 60, N = 200, decision tree with 10 cross validation, decision tree with external test, AdaBoost with 10 cross validation and AdaBoost with external test when training classifiers with different sample sizes.

Comparisons between our proposed and the baseline algorithms can be visualized directly in the following figures, figure 2, figure 3 and figure 4. These three figures indicate performances of different algorithms based on the same number of labeled training data.

We next compared the prediction accuracy with Support Vector Machine, which is the state-of-the-art algorithm for protein subcellular localization. Due to time constraint, we did not consider different values of labeled instances when training the SVM classifier, we used the 7,579 labeled instances and did a ten-fold cross validation. We tuned the *γ *parameter in RBF kernel, which is a typical setting in protein subcellular localization, and the different values of *γ *will undoubtedly affect the overall prediction accuracy. The experimental results are shown in Table 6.

**Table 6:** Performance of SVM

*γ*	Prediction Accuracy
0.00001	0.5865
0.00002	0.6162
0.00004	0.7050
0.00008	0.7524
0.0001	0.7661
0.0002	0.7807
0.0004	0.7957
0.0008	0.7887
0.001	0.7772
0.002	0.7279
0.004	0.6048
0.008	0.4185
0.01	0.3666
0.02	0.2827
0.04	0.2577
0.08	0.2560

From the results, we can see that SVM could almost achieve a 80% accuracy when *γ *is set to 0.0004, and typically the prediction accuracy is between 75% and 80%. However, as shown in our CoForest approach, the prediction accuracy can be increased to 85% when we are using only 3000 labeled instances for training, thus, by using about 40% of labeled instances, one can achieve a 10% performance increase than the state-of-the-art algorithms. This result is very promising.

## Conclusion

In this paper, we present a semi-supervised learning approach to solve protein subcellular localization problem. One particular feature of protein subcellular localization is that a large amount of unlabeled protein sequences are available but no literature tries to make use of these unlabeled instances. We used the CoForest algorithm and the large number of unlabeled protein sequences for predicting protein subcellular localization. Experimental results show that we can achieve more than 10% accuracy increase than SVM and moreover, we used only about 30% labeled instances to achieve this accuracy.

There are several possible directions for future research into this CoForest framework. The performance of CoForest may be better enhanced when we incorporate the active learning framework into CoForest, i.e. we could extract more useful information by selecting some representative unlabeled instances, instead of randomly choosing the unlabeled instances. Another possible solution is to further incorporate the transfer learning framework into this approach, where the distribution of unlabeled data may not follow the overall distribution of labeled data. Using a semi-supervised transfer learning approach may further improve the prediction accuracy.

## Methods

### Related work

In this paper, our proposed approach is based on the co-training paradigm, which is a very important algorithm in semi-supervised learning. Also, we exploit the ideas from ensemble learning to help improve the overall accuracy. In the following, we briefly introduce some related work in semi-supervised learning and ensemble learning.

Machine learning, or classification in particular, is concerned with fitting a function that maps a pattern to its corresponding class label based on prior knowledge and a set of features describing the pattern. For a traditional two-class classification problem, we are given a set of samples, i.e. a number of input vectors **x**_*i *_∈ ℝ^*d*^(*i *= 1, 2,..., *N*) with corresponding labels *y*_*i *_∈ {+1, -1}(*i *= 1, 2,..., *N*), where *N *is the number of labeled instances and *d *is the dimension cardinality of each training instance (that is, the number of features). The goal of a learning algorithm is to construct a binary classifier or a decision function which takes a new **x **as input and derives a corresponding label *y *∈ {+1, -1} based on the given labeled data. Typically, features are manually chosen to quantitatively describe each training instance or extract the most important values that can distinguish one class with another. From the view of statistical machine learning, experimental results usually show that the larger the *N *is, the better the overall prediction accuracy will be. As mentioned in the last section, manually labeling the data is a time-consuming task. There exists a large amount of unlabeled proteins, which traditionally are not taken into account in overall prediction. However, we think this is a mistake.

In traditional classification, all training data should be labeled before learning and the learned classifiers depend on these labeled data. When a large portion of unlabeled data are also available, a new opportunity is presented to improve the learning performance. An effective approach that has been used by machine learning researchers is the *semi-supervised learning framework*, where an initial hypothesis is first learned from the labeled data and then this hypothesis is refined, using the unlabeled data by some automatic labeling strategies, in several iterations.

There have been many approaches or algorithms that fall into the semi-supervised framework. Interested readers can refer to Zhu's survey on semi-supervised learning [44] for a comprehensive explanation about what semi-supervised learning is and some latest results.

Typical semi-supervised algorithms include the EM algorithms to estimate the parameters of the generative model and the probability of unlabeled examples in each class [45]; transductive inference for support vector machines [46,47], and so on.

The co-training paradigm is one of the early proposed framework that was well studied and developed [48]. In co-training, two classifiers are trained on two sets of attributes/features respectively. Each classifier will choose to label some unlabeled data for which they feel they are most "confident" with. These newly labeled examples are then added to the labeled training set of the other classifier. After that, each classifier is retrained using the augmented labeled data set, hoping that the "most confident" instances labeled by the other classifier will improve the generalization ability of the classifier learnt in this iteration. This process is repeated till converge is reached, or the difference in the classifiers learned in previous two rounds is relatively small. Co-training has been successfully applied in many applications, including statistical parsing [49], visual detection [50], etc.

Therefore, we believe it would be interesting to apply semi-supervised algorithm based on the co-training framework to the problem of protein subcellular localization. To our best knowledge, there has been no work that tries to solve the protein subcellular localization problem via a semi-supervised learning approach.

Ensemble learning is a very important machine learning framework that was usually explained as "wisdom of the crowds". In ensemble learning, multiple learners are trained and then their predictions are combined in order to make more accurate predictions. Experiments in many real-world datasets across a large number of domains show that ensemble learning can effectively improve the accuracy or generalization ability of many classifiers.

An ensemble learning algorithm usually has two steps, in which the first is to generate multiple classifiers and the second is to combine their predictions. Current trends tend to categorize ensemble learning algorithms in two categories, considering whether they generate the classifiers in a parallel way or a sequential way.

For the first category, where the multiple classifiers are generated in a parallel way, some representative algorithms include Bagging [51], which generates each classifier based on a training set bootstrapped from the original training set, this generating process can be done in a parallel way since different bootstrapping process do not affect each other. The predictions of these classifiers are combined using a majority voting. Other algorithms that fall into this category include stacking predictors [52], random subspace [53], random forest [54], etc.

For the second category, the most important and representative algorithm is AdaBoost [55], which sequentially generates a number of classifiers. The subsequent classifiers are targeted on the misclassified examples by the former classifiers.

Ensemble learning has been successful in many fields, including the protein subcellular localization problem. Recently Shen et al. [1] presents an ensemble learning algorithm for protein subcellular localization. Our approach combines semi-supervised learning and ensemble learning in hopes of much better prediction results for the biological problem.

### Proposed approach

In this paper, we use a new co-training style algorithm that was first proposed by Li and Zhou [56] which extends the co-training paradigm by an ensemble algorithm named Random Forest [54].

We let *L *and *U *denote the labeled set and unlabeled set. In co-training, two classifiers are trained from *L *and then each of them selects the most confident examples in *U *to label, from their own classifying function or separating hyperplane, respectively. Thus, an important part of co-training lies in how to estimate the confidence of prediction, in other words, how to estimate the confidence of an unlabeled example.

In Li and Zhou's proposed Co-Forest algorithm [56], an ensemble of *N *classifiers denoted as H* is used in co-training instead of two classifiers. In this way, we can estimate the confidence of each classifier efficiently. If we want to consider the most confidently labeled example by a certain component classifier of the ensemble *h*_*i*_(*i *= 1, 2,..., *N*), we use all other component classifiers except *h*_*i*_, called the *concomitant ensemble *of *hi *and denoted by *H*_*i*_. Therefore, the confidence of labeling can be computed as the degree of agreements on the labeling, i.e. the number of classifiers that agree on the label assigned by *H*_*i*_. The overall idea of CoForest is to firstly train an ensemble of classifiers from labeled dataset *L *and then refine each classifier with unlabeled data by its concomitant ensemble.

More specifically, in each learning iteration round of CoForest, the concomitant ensemble *H*_*i *_will test each example in *U*. If the number of classifiers that agree on a particular label exceeds a pre-defined threshold *θ*, the unlabeled example, labeled with this newly assigned label is copied into the newly labeled set ${L^{\prime }}_{i}$ Then for this round, set *L cup*${L^{\prime }}_{i}$ is used for refining *h*_*i *_in this iteration. Note that the unlabeled examples are not removed from *U*, so they might be selected by other *H*_*j*_(*j *≠ *i*) in the following iterations. One problem that may affect the overall performance of CoForest is that all the unlabeled data whose prediction confidence that are above *θ *will be added to *L*_*i*_, thus making *L*_*i *_rather large in the future. But in case the learned classifier cannot represent the underlying distribution, such a huge amount of labeled data will indeed hurt the performance, instead of helping the prediction accuracy. This phenomenon was discovered in several semi-supervised learning algorithms. Inspired by Nigam et al [45], CoForest also assigns a weight to each unlabeled example. An example is weighted by the predictive confidence of a concomitant ensemble. This approach makes the influence of *θ *insensitive, even if *θ *is small, the influences of examples with low predictive confidence can be limited.

In the CoForest algorithm, *N *random trees are firstly initiated from the bootstrapped training set from the labeled set *L *to create a random forest. Then in each iteration, each random tree will be refined with the newly labeled examples by its concomitant ensemble, where the confidence of the labeled example exceeds a certain threshold *θ*. This method will reduce the chance of the trees in a random forest being biased when we utilize the unlabeled data.

For detailed descriptions of CoForest algorithm, interested readers could refer to [56] for details.

## Competing interests

The authors declare that they have no competing interests.

## Authors' contributions

Qian XU and Derek Hao Hu identified the data set, conducted the experiments, and analyzed the experimental results. Hannah Hong Xue, Weichuan Yu and Qiang Yang designed and directed the research. All author worked on the manuscript. All authors read and approved the final manuscript
